# Helicobacter pylori in early childhood and asthma in adolescence

**DOI:** 10.1186/s13104-020-04941-6

**Published:** 2020-02-18

**Authors:** Kjetil K. Melby, Karin L. Carlsen, Geir Håland, Helvi H. Samdal, Kai-Håkon Carlsen

**Affiliations:** 1 Department of Microbiology, Oslo University Hospital, 0035 Oslo, Norway; 2 Division of Pediatric and Adolescent Medicine, Oslo University Hospital, 0407 Oslo, Norway; 3 Faculty of Medicine, Institute of Clinical Medicine, University of Oslo, Oslo, Norway

**Keywords:** Anti *H. pylori* IgG antibodies, Asthma in adolescence, Cohort study, *H. pylori* carriage in children and adolescence, *Helicobacter pylori*

## Abstract

**Objective:**

An inverse effect of *Helicobacter pylori* (*H. pylori*) on the occurrence of asthma is debated and early acquisition of *H. pylori* may be important. We analyzed sera from 197 children from Environment and Childhood Asthma (ECA) study in Oslo for *Helicobacter pylori* (*H. pylori*) at 2 and 10 years, and symptoms and signs of asthma at 16 years of age.

**Results:**

While 16.4% of children who were *H. pylori* negative at 2 and 10 years had current asthma at 16 years, none of the 12 children who were *H. pylori* positive at 2 years of age had asthma at the age of 16 years, regardless of *H. pylori* status at 10 years. This trend for less current asthma in children who were *H. pylori* positive at 2 years compared to persistent or transient negative status at 10 years was not statistically significant, probably due to low number of *H. pylori* positive children at 2 years of age. Acquisition of *H. pylori* in school age did not appear to influence the risk of current asthma. Much larger prospective studies are probably required to document whether or not early *H. pylori* infection may be involved in the risk of asthma development in later childhood.

## Introduction

The role of *Helicobacter pylori* (*H. pylori*) in asthma and atopy development is debated [1–3]. Some studies report reduced risk of asthma in children with IgG antibodies against *H. pylori* (*H. pylori* positive) [4, 5], while a meta-analysis of 770 cases and 785 controls concluded with no significant association between asthma and *H. pylori* carriage [6], and a recent study suggesting that *H. pylori* infection diagnosed in adults above 20 years of age may experience an increase in the risk of adult onset asthma [7]. In the Environment and Childhood Asthma (ECA) birth cohort study in Oslo with a prevalence of current asthma of 13.7% at 16 years of age [8] we recently showed that presence of IgG antibodies against *Helicobacter pylori* (*H. pylori*) as well as IgG antibodies against *cagA* at 16 years of age was associated with the absence of current asthma [9], and that most cagA positive individuals had high levels of *H. pylori* IgG antibodies [9]. Cross sectional studies have demonstrated inverse associations with current asthma in 3–13 year-old children and adults [4] and a meta-analyses observed a weak, but significant inverse associations in children and adults [5]. Also, in Ethiopian children, *H. pylori* positivity was associated with reduced risk of “any allergic condition” at 6 years, while at 3 years, *H. pylori* positive children had non-significantly more often wheeze and significantly less atopic dermatitis than *H. pylori* negative children [10, 11].

As there is limited knowledge of the associations between *H. pylori* IgG in early childhood and asthma in adolescence, we aimed to examine if the presence of *H. pylori* IgG at 2 and 10 years of age was associated with current asthma at in adolescence.

## Main text

From the asthma enriched general population ECA study [8] we included all 197 children who attended the 2–10 and 16 year follow-up investigations and had *H. pylori* analyses performed at the time of sampling at 2 and 10 years. Serum was analyzed in a conventional Enzyme Immunosorbent Assay (EIA) for anti *H. pylori* IgG antibodies and the results were classified as positive, borderline or negative all according to the manufacturer’s instructions (Orion Diagnostica, Espoo, Finland). An estimate of quantity of anti *H. pylori* IgG was also recorded in keeping with the manual following the assay. Anti-*H. pylori* IgG: negative (< 17), borderline (17–22), positive (≥ 23), all given in arbitrary units (AU). Current asthma at 16 years of age was defined as a positive response to at least two of the following three structured interview questions within the last 12 months: wheeze or shortness of breath, asthma medications and a doctor diagnosis of asthma [12]. Risk of current asthma by *H. pylori* status was compared by the χ^2^ test and statistical significance was set to 0.05%. All analyses were done in IBM SPSS version 25.

**Fig. 1:**
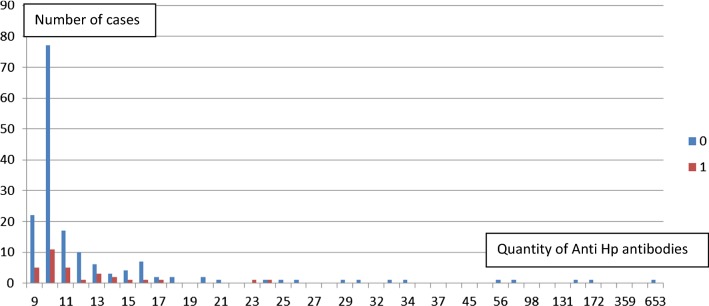
Serum levels of IgG antibodies against *Helicobacter pylori* at 10 years of age in a group of 197 children followed from 2 to 16 years of age in relation to presence (1 = Red) or absence (0 = Blue) of current asthma at the age of 16. The two children with current asthma and positive Hp IgG at 10 years were Hp negative and Hp borderline at 2 years, respectively. Anti-Hp IgG: negative (< 17), borderline (17–22), positive (≥ 23). All given in arbitrary units (AU)

Twelve children were anti *H. pylori* IgG positive by the age of 2 years, of whom five remained positive also at 10 years. The majority of *H. pylori* IgG negative children (94%) of children, who were *H. pylori* IgG negative at 2 years, were negative also at 10 years. None of the 12 children who had *H. pylori* IgG at 2 years of age had current asthma at 16 years regardless of *H. pylori* IgG status at 10 years, compared to 17% of the *H. pylori* negative children and one of the two children with borderline *H. pylori* positive at 2 years (Table 1) (p > 0.1 by χ^2^ test). Among the two children with current asthma at 16 years and *H. pylori* IgG at 10 years, one was negative and one borderline *H. pylori* IgG positive at 2 years of age. Their antibody levels to *H. pylori* were low in contrast to the other *H. pylori* positives patients who were non-asthmatic. At 10 years 17 children had positive *H. pylori* IgG antibodies with titers ranging 20–653 (Fig. 1).

**Table 1:** The Hp status at 2 and 10 years is shown in relation to current asthma (yes or no) at 16 years among 197 children

Hp status 2 years	Hp status 10 years	Current asthma 16 years N/n at 2 years (%)
Negative n = 183	Negative n = 172	30/183 (16.4)
	Positive n = 11	1/183 (0.6)
Borderline n = 2	Negative n = 1	0/2 (0)
	Positive n = 1	1/2 (50)
Positive n = 12	Negative n = 7	0/12 (0)
	Positive n = 5	0/12 (0)
Total		32/197 (16.2)

The finding that none of the *H. pylori* seropositive children at 2 years had current asthma in adolescence is in line with previous reports [3–5]. Our results are further supported by studies in mice showing that infection with *H. pylori* in the neonatal period prevented asthma development later in life. Importantly, primary infection with *H. pylori* in grown up mice, on the other hand, showed no such protective effect [13], in line with the recent increased risk of adult onset asthma in *H. pylori* infected individuals [7]. During the first years of life an adaption between the microbiome and the host takes place. The outcome of this interaction is regarded to be of substantial importance and ending in most cases in a delicate balance between the host immune system and the established microbiome. In developing countries the exposure to a microbiome containing *H. pylori* is common whereas in more affluent societies this is more seldom [14]. Our aim was to examine whether a significant inverse relationship between *H. pylori* and the occurrence of asthma could be demonstrated in our cohort. It is most likely that the microbiome/diet in a Nordic setting differs from a variety of African environments in which the carriage rate of *H. pylori* is higher. The presence of *H. pylori* in the Scandinavian communities at large is low [14]. Interestingly, none of the children with high levels of anti *H. pylori* antibodies suggesting brisk inflammatory response to *H. pylori* had current asthma. Whether other species and other microbiomes such as the skin microbiome may have a decisive role as a player in the control of the immune response resulting in a lower frequency of atopy and/or asthma as suggested by Finnish studies remains to be settled [15, 16]. Our results do not statistically support the hypothesis that there is an inverse relationship between the presence of *H. pylori* suggesting that early presence of *H. pylori* in this context is beneficial. However, the lack of asthma in high level *H. pylori* positive cases might justify further studies in areas where the prevalence of *H. pylori*, preferably carrying cagA, is higher. A real difference in occurrence of asthma and atopy was reported in the Karelian area with a Finnish and Russian area having similar climatic condition. Interestingly, the difference observed, i.e. low incidence of asthma and atopy in Russia compared to the Finnish sector, suggests that other factors, possibly related to differences in socioeconomic conditions, may in part explain these observations [15]. Furthermore Miftahussurur et al. reported that variation in carriage rate of *H. pylori* was not inversely related to asthma [2]. In conclusion, adolescent current asthma was not observed in any of the 12 *H. pylori* seropositive children at 2 years of age, regardless of *H. pylori* positive or negative status at 10 years in this prospective birth cohort study with a prevalence of current asthma of 13.7%. This may indicate that acquisition of *H. pylori* per se or a microbiome with presence of *H. pylori* at the age of 2 or less might prove beneficial.

## Limitations

Our results do not statistically support the hypothesis that there is an inverse relationship between the presence of *H. pylori* suggesting that early presence of *H. pylori* in this context is beneficial. Much larger prospective studies are probably required to document whether or not early *H. pylori* infection may be involved in the risk of asthma development in later childhood.

## Data Availability

Enquiries as to availability of data and material in this cohort should be addressed to KCLC.

## Abbreviations

ECA study: Environment and Childhood Asthma (ECA) birth cohort study in Oslo
*H. pylori*: *Helicobacter pylori*
*H. pylori* positive: Child with IgG antibodies against *H. pylori*
